# Using Fixation-Related Potentials for Inspecting Natural Interactions

**DOI:** 10.3389/fnhum.2020.579505

**Published:** 2020-11-05

**Authors:** Dennis Wobrock, Andrea Finke, Thomas Schack, Helge Ritter

**Affiliations:** ^1^Center of Cognitive Interaction Technology CITEC, Bielefeld University, Bielefeld, Germany; ^2^Neuroinformatics Group, Faculty of Technology, Bielefeld University, Bielefeld, Germany; ^3^Neurocognition and Action Group, Faculty of Psychology and Sports Sciences, Bielefeld University, Bielefeld, Germany

**Keywords:** fixation-related potentials, brain-computer interface, multi-modal system, interaction analysis, event-related potentials, eyetracking

## Abstract

Brain-Computer Interfaces (BCI) offer unique windows into the cognitive processes underlying human-machine interaction. Identifying and analyzing the appropriate brain activity to have access to such windows is often difficult due to technical or psycho-physiological constraints. Indeed, studying interactions through this approach frequently requires adapting them to accommodate specific BCI-related paradigms which change the functioning of their interface on both the human-side and the machine-side. The combined examination of Electroencephalography and Eyetracking recordings, mainly by means of studying Fixation-Related Potentials, can help to circumvent the necessity for these adaptations by determining interaction-relevant moments during natural manipulation. In this contribution, we examine how properties contained within the bi-modal recordings can be used to assess valuable information about the interaction. Practically, three properties are studied which can be obtained solely through data obtained from analysis of the recorded biosignals. Namely, these properties consist of relative gaze metrics, being abstractions of the gaze patterns, the amplitude variations in the early brain activity potentials and the brain activity frequency band differences between fixations. Through their observation, information about three different aspects of the explored interface are obtained. Respectively, the properties provide insights about general perceived task difficulty, locate moments of higher attentional effort and discriminate between moments of exploration and moments of active interaction.

## 1. Introduction

Brain-Computer Interfaces (BCI) are defined as a special subclass of Human Computer Interfaces. They take the user's brain activity as input to either actively generate commands or passively monitore the user's state. BCI primarily rely on the communication between two of its components: a user-side component and a system-side component (Graimann et al., 2010). The primary role of both components is to record data relevant to a monitored interaction. One component focuses on data originating from the user (e.g., brain activity) and the other on data from the system (e.g., currently displayed stimuli). To ensure adequate BCI performance, brain activity has to be analyzed relative to interaction-relevant moments of interest (e.g., input events or received feedback). This is done to correctly associate cognitive processes detectable in the brain activity with corresponding actions performed during interaction. Consequently, obtaining valuable insights into the interaction necessitates that the communication between these two BCI components occurs accurately and synchronously. To achieve this, both components are often specifically designed to work in very specific controlled scenarios (Tan and Nijholt, 2010), creating a precise but rather inflexible BCI. When stepping outside of these specific use cases, the information from one component provides little to no informative value to effectively investigate the new interaction.

User-side BCI components aim to record any user-related information (such as biosignals) during interaction. This is commonly achieved with arrays of sensors such as in Electroencephalography (EEG). Sensors are generally conceived to suit a wide scope of users, varying in physiognomy and expertise. They usually serve to exclusively acquire and transmit physiological signals, rarely physically influencing the underlying interaction situation they are used in. This aspect allows user-side components to be easily transferred between many experimental setups without much adaptation to conform to new BCI paradigms. As a result, the manipulated machine (and its interface) becomes the main locus of required adaptations to satisfy the necessary BCI system-side requirements, thereby putting the burden of producing and relaying relevant context information mainly on the system-side component. Practically, the system-side is required to produce triggers or stimuli to help contextualize data recorded by the user-side component. Unfortunately, this frequently involves completely overhauling the original machine interface to accommodate for the requirements. This necessity introduces a significant workload during implementation and is a sharp limiting factor for expanding a same BCI setup to a wider scope of uses.

Adaptation becomes even more extensive when relying on a particularly common yet informative BCI paradigm: the study of Event-Related Potentials (ERPs). With ERP, the user's brain activity is analyzed at the onset of certain types of events (Teplan et al., 2002). These commonly system-triggered events do not only provide the required context information for the data analysis but can also sometimes induce meaningful brain activity by presenting contingent stimuli (e.g., flashes of the P300 speller as a keyboard substitute; Farwell and Donchin, 1988). While considerably increasing the quantity and quality of information that can be extracted from the raw data, implementing ERP-based system-components can also strongly restrict interaction. Indeed, ERP-based BCI relies on the reaction to events (e.g., visual or auditory stimuli such as beeps or light flashes; Holcomb et al., 1992) which are not traditionally present in most common interfaces. When adding these events, the nature of the underlying interaction is thus changed from its original process, likely altering the scope of cognitive processes that are observed with the BCI. If BCI is used to investigate an existing human-machine interaction in greater detail, information obtained through common ERP paradigms thus may often not be valid for describing the initial interaction.

**Figure 1:**
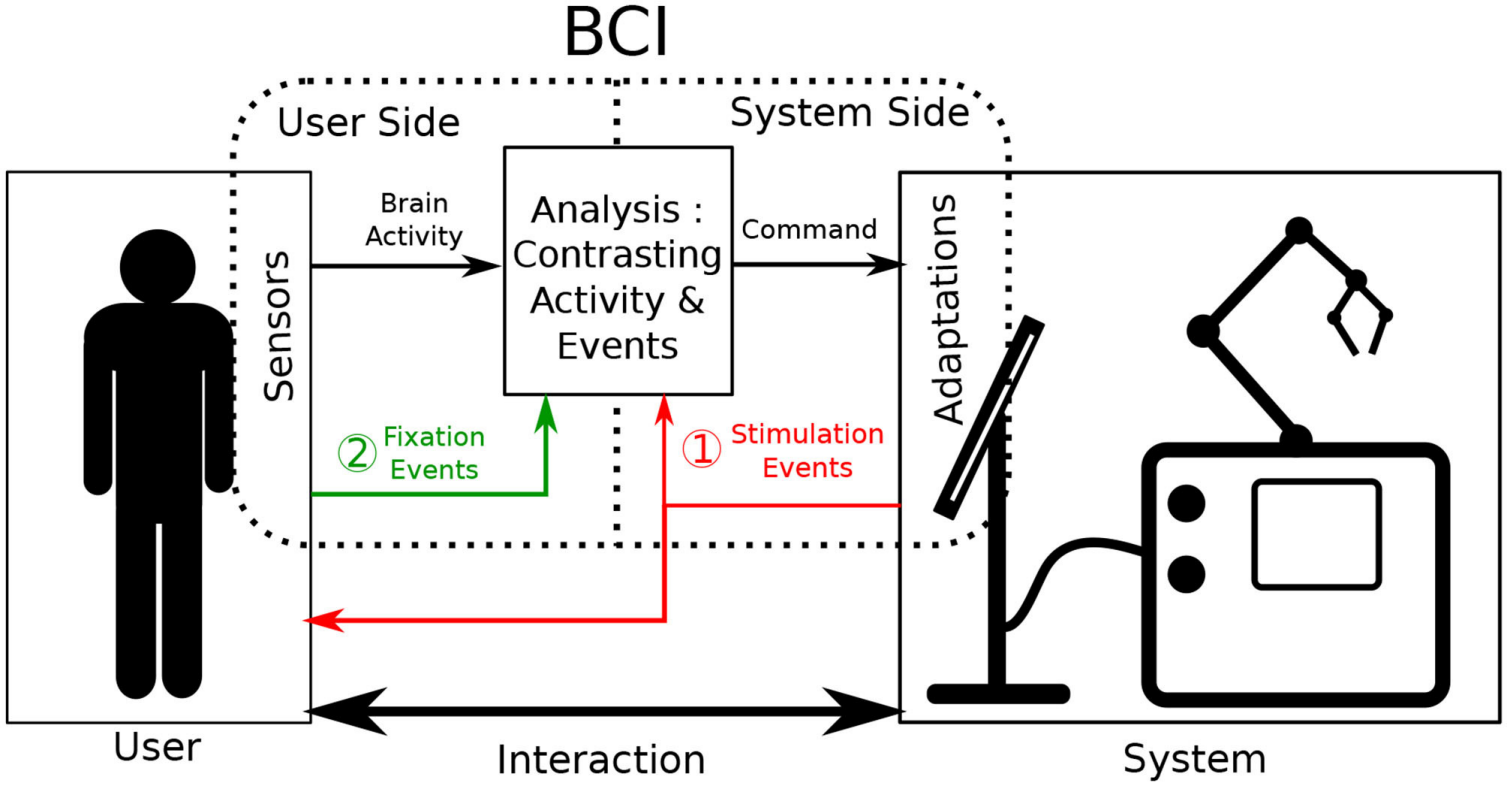
Illustration of the functioning of Event-related potential Brain Computer Interfaces during interaction. Sensors are placed onto the user and adaptations are made on the system to augment interaction. The brain activity of the user is contrasted and analyzed as it relates to events. Classically (case 1 in red) these events are produced by system adaptations. In this work, we investigate the acquiring of events via sensors such as eye tracking (case 2 in green).

Fixation-related Potentials (FRPs) are a subcategory of ERPs that rely on eye-fixation based events to give context to brain activity (Baccino and Manunta, 2005). Consequently, user-side components then also have to host eye-tracking technology. By their nature, eye movements are user-generated and occur separately from any system while still being influenced by presented stimuli (Mills et al., 2011). Accordingly, using eye-motion as events in an ERP-based BCI means that context-giving events are produced by user-side components themselves instead of system-side ones (see case 2 in Figure 1). BCI system adaptation requirements can thus be partially circumvented, working toward a more balanced distribution of adjustment burdens of the two BCI sides.

However, FRP-based BCIs are still rather novel and need a general approach on how their potentials can be effectively and efficiently tapped to provide meaningful insights about the ongoing interaction. Indeed, utilizing eye fixation as context events without knowing the contents of a fixated visual region may seem inefficient to gauge interaction. So far, FRP BCI is still a barely explored subject, mimicking classical ERP-based interfaces (Finke et al., 2016) and their system-side requirements. Nonetheless, their bimodal nature allows FRPs analysis to study interactions from new and multiple different angles. In this work, we outline three of these different ways to utilize FRPs, their properties and sensors used to acquire them, to reduce reliance on system-side information and still access valuable information about the interaction. Both EEG and Eyetracking have already found their application in interaction analysis (Jacob, 1995; Ewing et al., 2016) but have to be examined together in greater detail to determine what additional information can be extracted.

The goal of this work is to investigate the informational value a bi-modal BCI, combining EEG and Eyetracking, can offer for assessing an interface without relying on system-side information. In the remaining part of this contribution, we will discuss different properties which can be analyzed through this BCI and what information about the interaction they hold. Starting by using Eyetracking individually, gaze patterns can be assessed which correlate with the general task difficulty relating to interaction. Secondly, using a bi-modal analysis of FRPs, the amplitude of early potential peaks can be used as an indicator for the cognitive attentional cost. With these stable peaks, user-fixated regions can be probed for their density and relevance of visual information. Lastly, utilizing a fixation-bound segmentation of the EEG signal and analyzing the relative frequency band power of the segments, discrimination between moments of exploration and moments of active manipulation becomes possible without system-side information.

## 2. Related Work

### 2.1. Context Information in BCI

Brain-Computer Interfaces can broadly be divided into two major categories: active and passive interfaces. Active interfaces allow their users to perform a set of direct and intentional actions on the system while bypassing classical interaction tools (e.g., mouse, keyboard, or general muscle movement). Passive interfaces, on the other hand, monitor the user's brain activity continuously and modulate the underlying interaction accordingly, without requiring volitional input from the user (Tan and Nijholt, 2010). In either case, broadening the scope of usability of existing interfaces via this technology is a consistently pursued goal of BCIs. However, achieving this has proven to be difficult.

Active interfaces rely on thorough bi-directional communication between their user and the manipulated system. The user selects an interface option by completing trials of the BCI paradigm. The system subsequently answers with the corresponding feedback. Regular BCI interaction is represented case 1 of Figure 1. Attaching active BCIs to already existing systems often implies that the communication from system to user remains identical. On the other hand, the user only communicates to the system through BCI-related commands, which is the defining feature of active BCI. This interaction format based on a rigid interlocking of two communication streams means that a high adaptation burden on the system side can hardly be circumvented, as accurate and goal-driven communication is essential.

In passive interfaces, communication does not need to be as controlled as explicit and direct interface control is not provided or required. Passive BCIs still operate as shown in Figure 1, commonly using case 1. However, as opposed to active BCI, interaction is performed bidirectionally as it would be naturally: both in what the system gives the user and in what the user inputs into the system. In parallel, the BCI continues to operate by producing additional commands aimed to adapt interaction. However, for this BCI information to be useful to the interaction, the interaction context still needs to be monitored. This is commonly done by adapting the system-side component. For our application however, in the spirit of minimizing system-side adaptation, context information gained from the user-side component should be maximized.

User context-information can be gathered indirectly through the monitoring of physiological signals as represented by case 2 in Figure 1. This has been demonstrated by continuously probing the EEG stream for a general arousal level during a long task (Lim et al., 2014). This approach is however very limiting in the amount of context-information it provides, as it only gives clues about the users general cognitive state. Continuous EEG monitoring doesn't allow to determine notable, more localized, interaction moments on its own. To improve upon it, additional physiological signals have been used as information supplements in a variety of BCI studies (Li et al., 2016). Some of the more commonly used signals include Electromyography (EMG) as well as Galvanic Skin Response (GSR) (Müller-Putz et al., 2015). These modalities have shown to reliably improve predictive accuracy of BCIs (Nijholt et al., 2011), but still only provide general insights into the users state. They fail to supply precise and momentary information about any performed action.

For such an approach, another promising physiological modality can be used: eye movement data, which are recorded by Eyetracking. Eyetracking itself has already frequently been employed to assess and characterize interaction in a wide scope of interfaces (Goldberg and Kotval, 1999). Eye movements have been shown to be influenced by user intention and the general task they pursue (Hayhoe and Ballard, 2005). Their utilization, while strongly dependent on the use case, can be applied to estimate certain specificities of the interaction (Henderson et al., 1999) (e.g., task-relevant visual regions), giving information about the user, as well as their surroundings. Furthermore, as opposed to continuous modalities such as GSR, EMG, or EEG, eye movement can be decomposed into discrete events (e.g., fixations and saccades) which help to segment and analyze recorded brain activity as ERP. Combining EEG and Eyetracking may thus allow to combine the strength of both approaches to gain a holistic view of the interaction. So far, the combination of these modalities has been exploited to improve the accuracy of existing BCIs (Protzak et al., 2013), but has only rarely been used in general interface analysis. The use of Fixation-related potentials may allow for these kinds of analysis.

### 2.2. Fixation-Related Potentials

Fixation-related Potentials have only recently been studied and can be observed through combined analysis of EEG and Eyetracking recordings. This type of ERP is time-locked to the onset of an eye fixation. This characteristic aligns these events with the underlying cognitive processing which occurs during visual exploration (Holmqvist et al., 2011). This subsequently allows to inspect said cognitive processes as they relate to the examined visual information.

These potentials have so far been examined in psychological studies investigating their informative value in simple visual tasks. This includes the identification of target objects (Brouwer et al., 2013), reading tasks (Takeda et al., 2001), or face recognition (Kaunitz et al., 2014). In most of these cases, FRPs have been studied in very controlled environments, proposing artificial and curated stimuli to the users during interaction. Yet, by their nature, these potentials lend themselves to an enhanced evaluation of brain activity in a wide variety of more natural scenarios (Dimigen et al., 2011). Indeed, the additional recording of eye movements allows for the monitoring of where and how the user attention is allocated with respect to the manipulated system (Luck et al., 2000).

FRPs have so far rarely been used in an actual BCI setting. When they are utilized, they serve as a new medium through which commonly used BCI paradigms are explored anew and extended. This includes paradigms comparable to the P300 oddball and N400 studies (Finke et al., 2016; Wenzel et al., 2016; Coco et al., 2020). However, these uses still require extensive system context information to function. Considering the established links between gaze direction and subject task, FRPs are also likely contain valuable information about a currently performed interaction and may thus overcome these limitations. Indeed, it has been shown by Shishkin et al. (2016), that FRPs allow to identify the intention of users from fixations, improving interaction for active BCI. By refining analysis of FRPs in the context of a passive BCI, a better understanding of interactions may be achieved. Additionally, the setup required to measure FRPs enables other types of analysis, such as using EEG and Eyetracking technology separately, and thus increasing the amount of potential information. Through this approach, methods may be found which can be used to broaden the usability of BCIs.

The guiding research question is: Can the data gathered through the proposed EEG and Eyetracking setup, including resulting FRPs, provide new insights in helping to understand common interaction? To investigate this aspect, datasets are required with which this setup and the data it provides can be studied in a naturalistic interaction environment. To increase the relevance and transposability of the findings to other interactions, the considered interaction should resemble interactions commonly occurring in real life. This means that they include phases where the interface is probed for information by the user and phases of active manipulation. The recorded interaction would have to feature both phases of active and passive interaction while still providing accurately labeled data as ground truth for a detailed analysis.

## 3. Materials and Methods

To explore the informative capabilities of an EEG and Eyetracking BCI in natural interaction, paradigms are required which are representative of natural interaction. However, these paradigms also need to remain sufficiently controlled to ensure the acquired data is meaningful labeled for subsequent validation. The proposed study was designed to allow for thorough analysis of FRP while maintaining a generic approach to interfaces which can be extrapolated from. A simple visual search task was chosen. It contained the two fundamental aspects of interaction: exploration of an environment as well as input of commands for selection made within this environment. This dataset allows for FRPs to be easily extracted and accurately analyzed as they relate visual information or action performed during interaction. The study was performed in a completely controlled computer-generated environment to ensure the correct logging and labeling of all data.

### 3.1. Task

**Figure 2:**
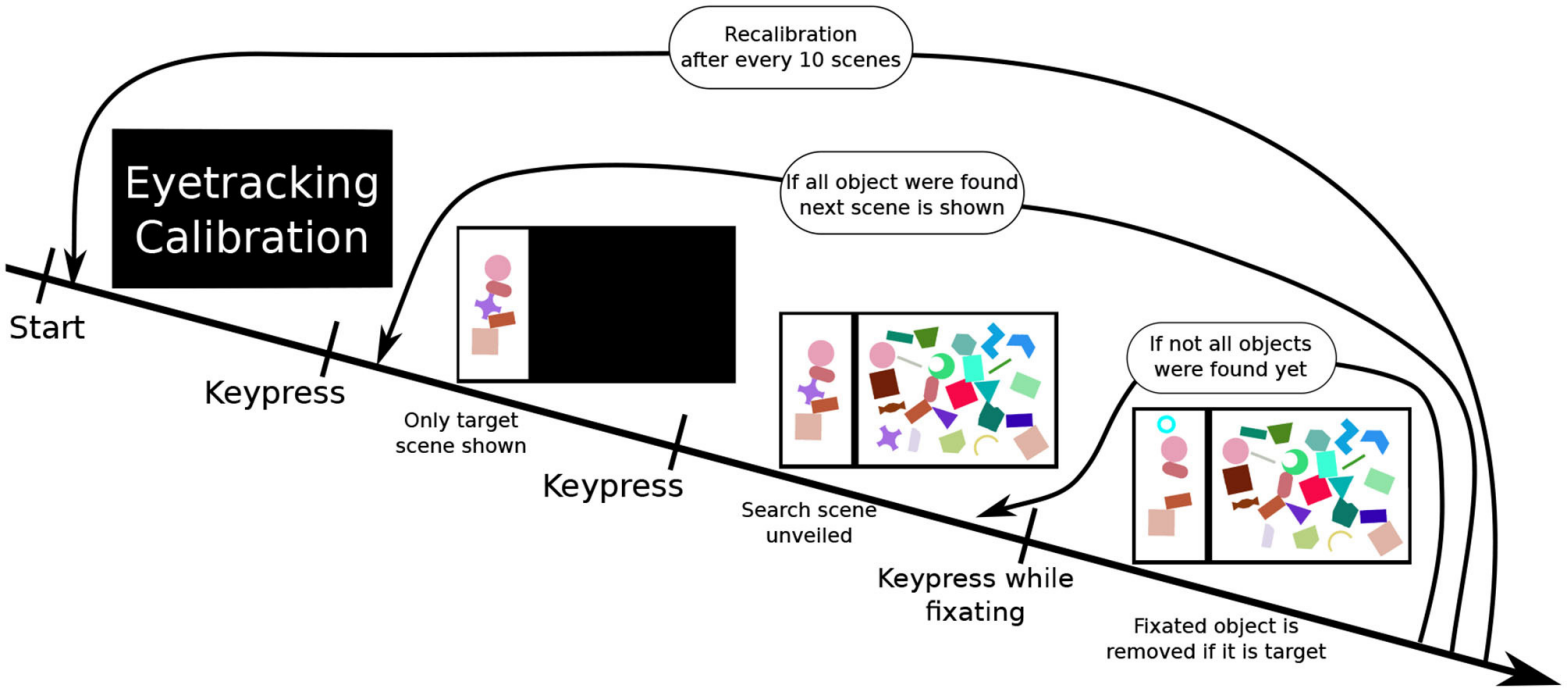
The experiment procedure: First, a calibration of the eyetracker is performed. Once completed, the participant performs a keyboard press showing a scene. At first, only the target scene is shown. Upon performing another keyboard input, the search scene is revealed. The fourth image illustrates a correct selection, the participant performed pressing space while fixating on a target object in the search scene. This removes the shape from both scenes (here the purple cross) and displaying a feedback image (cyan circle) in the upper left corner. This process is repeated until all target objects are removed. After this, the next search scene is displayed.

Participants were presented with a scene on a 1,920 × 1,080 pixel (24 inch) computer screen, which was split into two sections: a smaller “target scene” on the left side and a larger “search scene” on the right side. The search scene was initially hidden. Both parts of the whole scene contained a variety of geometric shape. The target scene contained between three and five non-overlapping shapes, while the search scene contained a much greater number of sometimes overlapping shapes (as illustrated in Figure 2). Subjects had to locate the shapes presented in the target scene within the search scene. Practically, participants were told to fixate an individual target shape (as measured with the Eyetracker) in the search scene and simultaneously execute a keyboard input (pressing the space key) to validate their selection. A short feedback sound, either a soft positive bell sound or harsh negative buzzer sound, was played depending on the validity of their selection. Concurrently, a small feedback image was also shown in the upper left part of the screen. If the selection was done correctly, the target shape was removed from both scenes. To ensure participants remained vigilant during the entire experiment, in rare cases (8% of scenes) some target objects shown in the target scene were missing in the search scene. In these cases, the participants had to perform the same selection procedure (i.e., simultaneous fixation and keyboard press) on the shape in the target scene, which is missing in the search scene. A scene was completed when all target objects were either selected or 3 min after the unveiling of the search scene.

### 3.2. Stimuli

**Figure 3:**
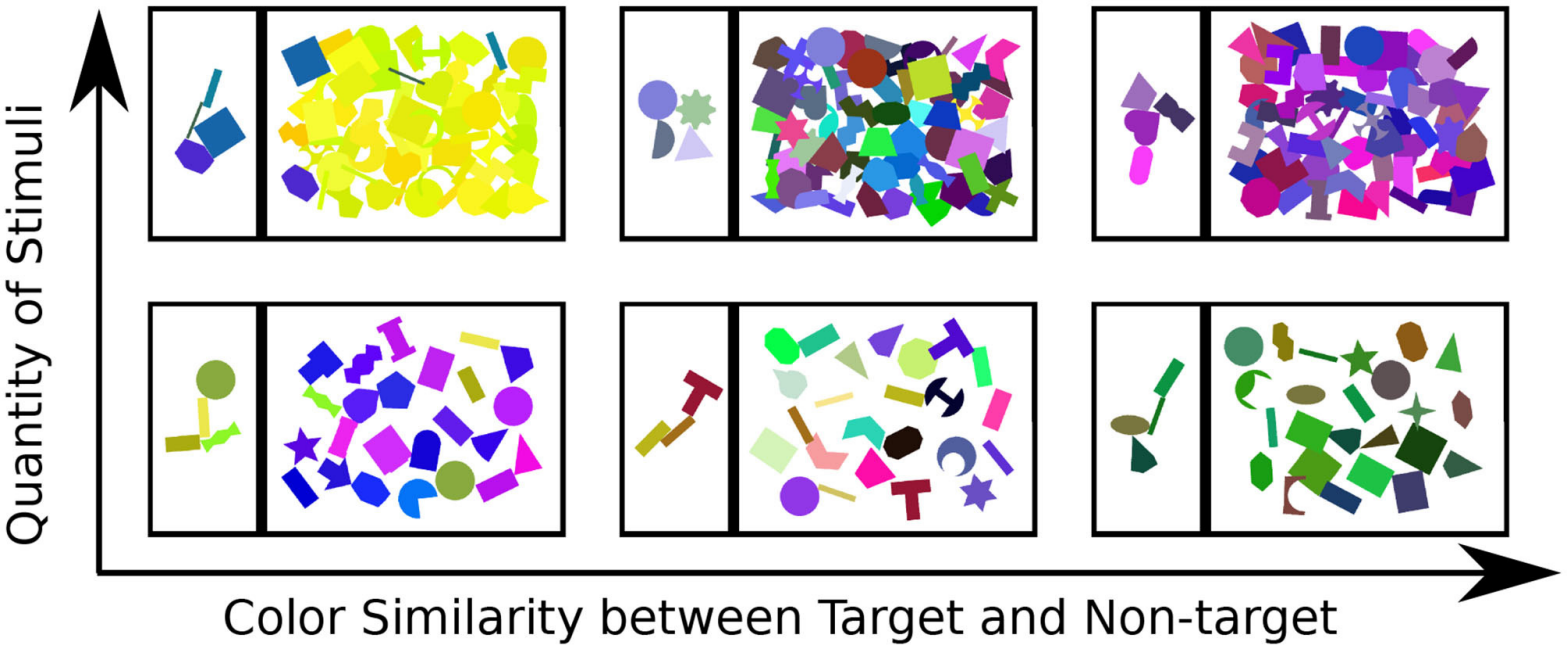
Examples for each of the six types of stimulus scene presented to the participant: top to bottom show the variation in quantity of objects, left-right illustrates the color similarity between target and non-target objects.

The participants were presented with a series of 138 scenes. These scenes varied in terms of color similarity between target and non-target objects (in three categories: high, medium, and low similarity) and in terms of number of objects (in two categories: few and many objects) present (which includes how strongly they overlapped) in the search scene (see Figure 3). Twenty-three pre-constructed scenes of each condition were shown to the subject in a random order. To introduce participants to the task, a small tutorial session featuring ten additional scenes was presented prior to the actual recording session. This also allowed them to familiarize themselves with interface operation.

The objects presented within these scenes were abstract 2D geometric shapes. Their size was scaled to fit exactly within a bounding box of fixed dimensions. Shapes were randomly selected from a larger list during scene creation. Shapes were also randomly rotated before being placed in a scene. The positioned shapes could be both convex or concave, ranging from simple (such as squares or circles) to more complex forms (such as half-moons or arrows). A uniform color was applied to each individual shape with no contouring color. The distance in color D between any two of these shapes (here labeled i and j) was determined by Equation (1).

(1) $$\begin{array}{r} D_{ij}=\sqrt{2{\left(R_{i}-R_{j}\right)}^{2}+4{\left(G_{i}-G_{j}\right)}^{2}+3{\left(B_{i}-B_{j}\right)}^{2}} \end{array}$$

With *R*_*i*_,*G*_*i*_,*B*_*i*_ corresponding to the red, green and blue values respectively of a chosen color for a given shape i. This equation was chosen to approximate the human perception of color distances (Robertson, 1990). In scenes featuring “high” color similarity, the value of distance D was kept between a value of 0 and 200. This range changed to 200–475, and 475–765 to consider color similarity “medium” or “low,” respectively. In scenes featuring many objects, the centers of each shape were placed at a Euclidean distance of at least 100 pixels apart. In scenes featuring few objects, this minimal distance was increased to 170 pixels.

### 3.3. Apparatus

EEG activity was recorded through 16 electrodes positioned at the locations Fz, Cz, Pz, Oz, F3, C3, P3, F4, C4, P4, PO8, PO7, F7, F8, T7, and T8 respective to the 10-20 system (Acharya et al., 2016). Additionally, horizontal and vertical Electrooculography (EOG) was also measured using four further electrodes. Two g.tec g.USBAmp biosignal amplifiers were synchronized via g.INTERSync cable and used for the EEG recording (at 256 Hz). Eye movements were recorded using an LC Technologies Eyefollower Desktop Eyetracker. This Eyetracker (ET) has a location accuracy with <0.4 degree error. The Eyetracker was positioned below the interaction screen and calibrated through a 9-point screen calibration at the start of the experiment. The device was also recalibrated using the same procedure after every tenth scene was completed or between any two scenes if the subject deemed it necessary. Eye gaze samples were recorded with a frequency of 60 Hz for each eye in alternation (120 Hz total). Both devices were operated by two different computers whose clocks were previously synchronized. Due to their differences in sampling rate, comparing the timestamps of an EEG sample to an ET sample introduces an inaccuracy range of 2–4 samples. Participants were seated facing the screen at a distance around 80 cm. As a result, their foveal region during the study encompassed a circular screen region with a radius of about 80 pixels.

### 3.4. Participants

Twenty-one volunteers participated in the study. The participants were aged between 19 and 38 years (mean 26.5 ± 5.0, 14 female). All were recruited from the local student, university visitors, and staff population and were either paid for their expenditure of time or granted course credit. All participants had no known prior or current pathological neurological condition (based on self-report) and normal or corrected-to-normal vision. The experimental procedure and written consent form for this study were approved by the ethics committee at Bielefeld University, and adhered to the ethical standards of the seventh revision of the Declaration of Helsinki. All participants gave their informed written consent to participate in the study.

### 3.5. Pre-processing

Once a participant completed the entire 138 scenes, their data was saved. Every information presented to the participant was also logged with the respective timestamps. This included what stimuli were presented to the subject, moments of action (i.e., keyboard inputs), feedback shown, sounds played, and performed recalibrations of the Eyetracker. All pre-processing was performed offline. Fixation locations and onset times were extracted from raw eye-tracking data of the subject's dominant eye. The fixation detection algorithm registered a fixation when the subject's eye gaze remained in a 5 degree eye-rotation angle area for at least 100 ms. Code-wise, this translated to the gaze position remaining in a circular region of 80 pixels diameter during 6 Eyetracker frames. A fixation was considered over once the gaze jumped further than 80 pixel from the previous frame. Practically, the software provided by the manufacturer was used with these thresholds. This ensures an easy reproduction of the results. The EEG data were firstly filtered using a 0.1 Hz highpass and an 8th order Butterworth 45–55 Hz notch filter. This was done to remove low frequency artifacts as well as artifacts originating from device voltages.

Subsequently, artifactual components, resulting from eye, head, and other muscle movements, were removed from the signal using an Independent Component Analysis (ICA) performed on the EEG channels (Jung et al., 2000). Artifactual ICA components were identified and rejected using the Multiple Artifact Rejection Algorithm (MARA) (Winkler et al., 2015). The usage of MARA for artifactual component selections also facilitates the reproducibility of the results. The ICA and MARA calculations were performed using the EEGLAB Matlab Toolbox (Delorme and Makeig, 2004) with default parameters. Furthermore, due to desynchronization between recording modalities, the EEG and Eyetracker recording timestamps were adjusted using a resynchronization method developed to deal with static and linear offsets (Wobrock et al., 2019).

## 4. Results

To be relevant for examining interactions, information extracted from the combined analysis of EEG and Eyetracking needs to provide valuable information about the considered interaction. To remain useful across a wide variety of interfaces, the methods used to access this information need to be applicable in many different situations. Here, using the recorded data, three methods are detailed which provide insights without requiring system-side information, thus allowing to be easily applied to new interaction situations in the future.

### 4.1. Analyzing Recording Modalities Separately

EEG and Eyetracking technology have commonly been used individually to assess interfaces. Before using them conjointly, information from either modality can be inspected separately. Looking at single modalities is notably interesting for gaining information into more general aspects of interaction, such as overall satisfaction or difficulties (Tanriverdi and Jacob, 2000). These kinds of analysis are performed using EEG to attain indicators of general attention or wakefulness during interaction (Bonnet and Arand, 2001). The use of Eyetracking technologies notably makes the analysis of gaze patterns possible.

Indeed, Eyetracking, used independently, serves as a means for the probing of interfaces. It is particularly utilized when validating the usability of an interface layout (Goldberg and Kotval, 1999) providing information about which regions are most easily perceived and explored by the user. However, an adequate probing of the interface requires comparing gaze patterns to the displayed interface elements and information related to them. Classically, system-side adaptation would thus be required to access the precise location and role of each presented stimulus and determine which areas in the interface are of interest to the user. Instead, as we aim to avoid this requirement, more generic measurements taken from Eyetracking are used to identify the general difficulties the user might be facing.

**Figure 4:**
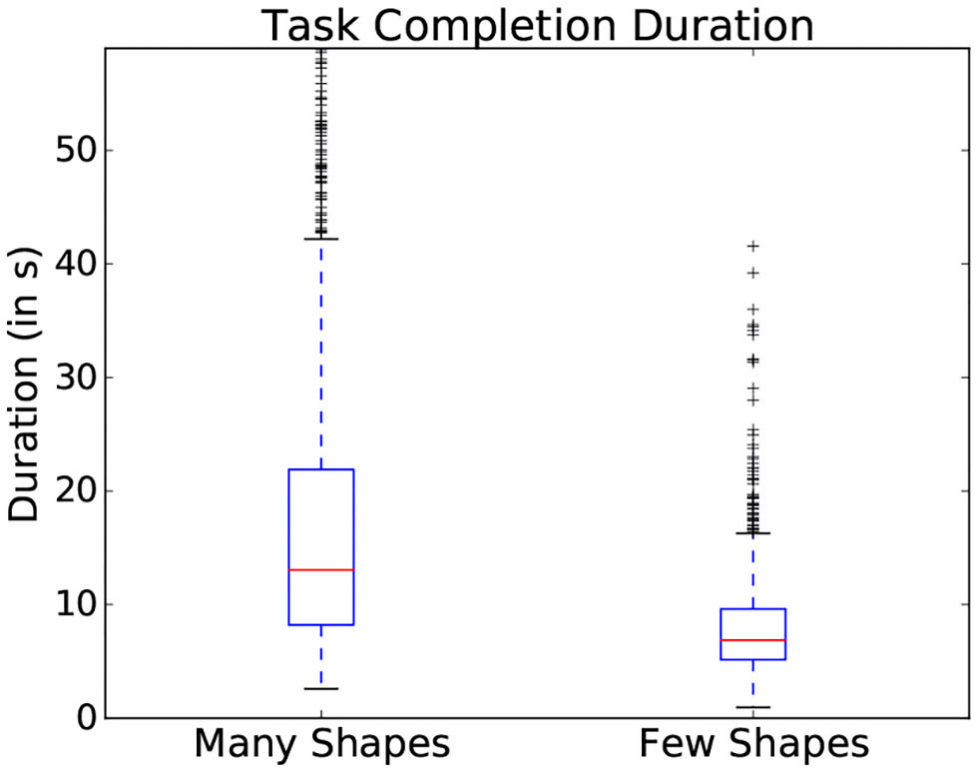
Variation of task completion duration depending on the number of objects present in a scene. Data from all participants is used in this figure.

Within the presented study, general difficulty was introduced using the two scene dimensions: the number of objects and the similarity in color between objects (as represented in Figure 3). These parameters showed an influence on the gaze patterns with which participants explored the scene. One common indicator of general difficulty is the time taken to complete a scene: the more objects were present within the scene, the longer the participant took to complete it and the more fixations were made (as shown in the boxplots represented in Figure 4). However, task duration, the number of fixations and their interpretation vary drastically depending on the studied interface and on the participant, making these measures prone to uncertainties on a task by task basis. This can notably be seen in the standard deviation shown in Figure 4, making it difficult to effectively use this metrics to assess interaction in most situations.

**Figure 5:**
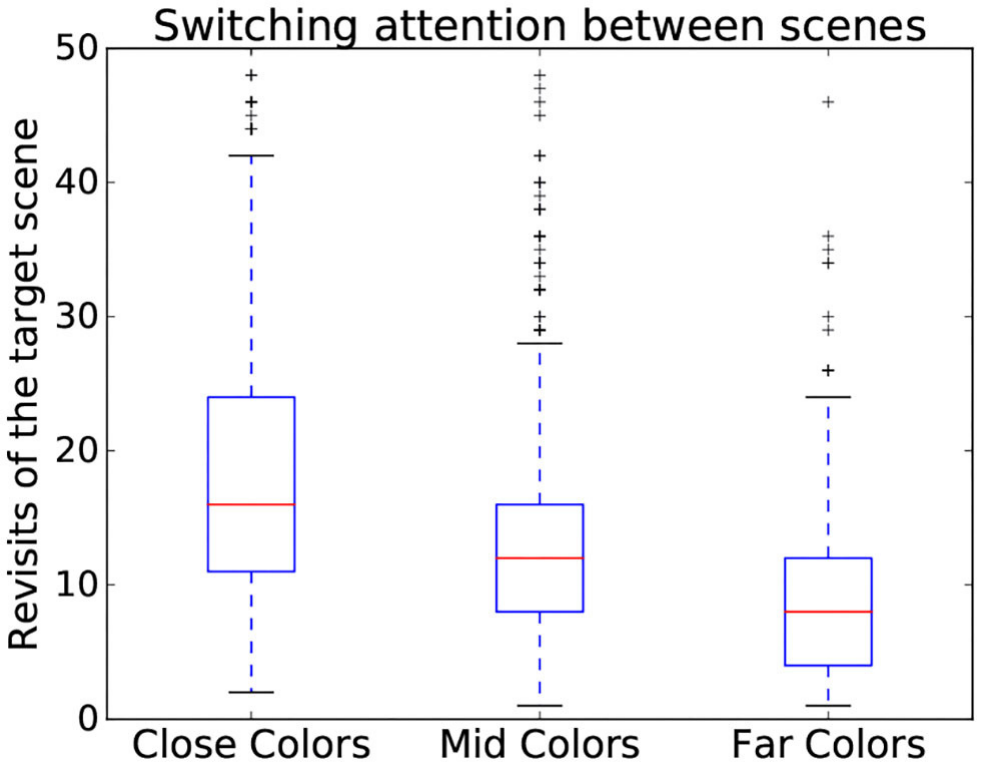
Variation of number of revisits of target scene depending on the color similarity present in a scene. Data from all participants is used in this figure.

Using time-independent measures can also serve to assess the interaction on a more abstract level. Indeed, time-independent measures also contain features useful for showing variation between different complexity categories (Zhang and Seo, 2015) even beyond the number and saliency of stimuli, such as the familiarity of the stimuli. This is the case here, where different patterns could be observed depending on scene complexity dimensions. Indeed, the more similar the colors, the more subjects tended to switch back and forth between the search and target scenes as seen in Figure 5. To expand on informative value of time independent measures, three gaze metrics have been chosen to assess difficulty as they encode relevant parts of the interaction:

- M1: the relative amount of long fixations (i.e., fixations longer than 250 ms; Hooge et al., 1998).
- M2: the relative number of distant saccades (i.e., saccades going outside the foveal region covered by the previous fixation).
- M3: the relative number of times the subject revisited previously fixated regions (i.e., fixation within the foveal region of any prior fixation).

The word “relative” refers to a percentage, such as the ratio of long fixations to all fixations done during one scene. The last two gaze metrics were extracted from the eyetracking recording by allowing the software to memorize the location of fixated regions during interaction. These metrics summarize certain aspects of the interaction in a time-independent manner effectively. Notably, in an abstract fashion, the ratio of distant saccades represents how often the participant switched their gaze between the target and search scene parts during manipulation.

**Figure 6:**
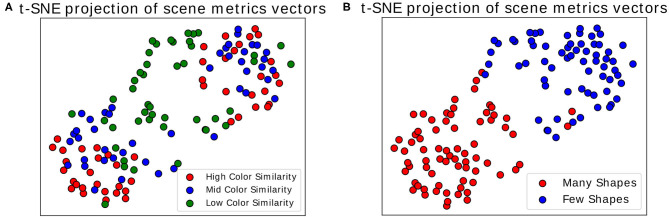
t-SNE projection on two dimensions. Each point represents a scene, represented through the three time-independent metrics. **(A)** Colors show the variation in color similarity between target and non-target objects. **(B)** Colors show the variation in number of objects in scenes. Only the data of one participant was used for these figures.

Once we calculated these metrics for every scene, we visualized the distribution of the three dimensional point cloud [with *M*(*i*) = (*M*1(*i*), *M*2(*i*), *M*3(*i*)), for each scene i, as a point] using the two dimensional projection resulting from the t-distributed Stochastic Neighbor Embedding algorithm (t-SNE) (van der Maaten and Hinton, 2008). In this visual projection of the distribution, differences between difficult and simple scenes can already be qualitatively observed (see Figures 6A,B). These visualizations make apparent that most classes appear easily separable, forming distinct clusters. Exception to this are the “medium” and “high” color differences, which mix these features together (as seen in the colors red and blue in Figure 6A). Properly associating a scene as belonging to one of these categories is subsequently performed through multiclass Fisher Discriminant Analysis (FDA) classification (Bishop, 2006). All three metrics were collected from the 138 scenes separately for each subject and used as features in this five-fold FDA classification (i.e., using four folds as training and one for testing, swapping, and repeating). Each fold contained the balanced amount of data from all categories of scenes (as defined in Figure 3). The classification aimed to gauge the separability these gaze metrics provide, to tell apart the different categories of presented scenes, and thus the scene composition variables modulating difficulty (see Table 1).

**Table 1:** Multiple linear classification utilizing the combination of three visual time independent features.

	**Classification accuracy**	**Chance level**
Discriminating number of objects	69.18% (±3.86)	50% (2 classes)
Discriminating color similarity	53.63% (±7.75)	33.33% (3 classes)
Discriminating all scene types	26.28% (±3.48)	16.67% (6 classes)

The results show that while good separability cannot be achieved through these metrics alone, above-chance level results still emerge when comparing gaze metrics coming from scenes with different difficulty dimensions. Comparing the classification scores between each other then allows to get an indication of the general difficulty a user faces during a scene without possessing any information about the stimulus layout. This information, particularly when using the t-SNE projection, makes this transposable method a possible starting point for obtaining general information about the type of task the user is performing, as it suggests where general difficulties may lie. Using the presented data as an example, scenes presenting high and medium color similarly were inspected in a similar fashion by the participants, indicating little perceptual difference between the two. For a more focused and in-depth approach to assessing interaction, the following analysis may utilize multiple modalities at the same time and restrict itself to more distinct moments occurring during interaction, e.g., by looking at well defined events occurring during interaction. As a promising candidate for such an approach, we consider in the next subsection the analysis of Fixation-Related Potentials.

### 4.2. Analyzing Fixation-Related Potentials

**Figure 7:**
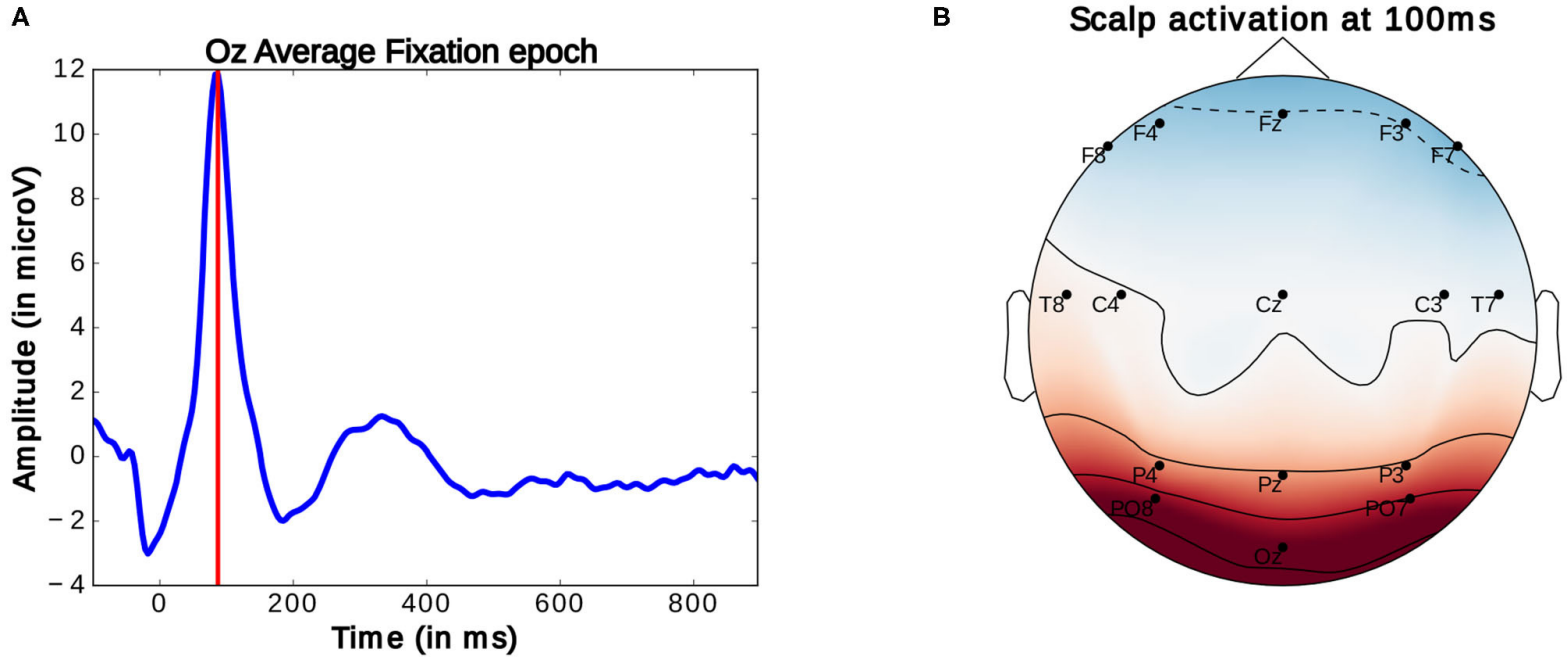
**(A)** Average Fixation related potential epoch profile recorded on the Oz electrode. The red line marks the location of P100 peak. **(B)** Scalp activation at the P100 peak. A redder color indicates a higher measure voltage at that location. The bottom central electrode is Oz. Only data from one participant was used in this figure.

Generally, in the study of ERPs, the continuously recorded EEG signal is analyzed at particular moments of interaction. These moments are marked by specific logged events which allow for the segmentation the signal into same length intervals, referred to as epochs. These epochs can then be compared. Here, for the observation of FRPs, the EEG signal is segmented into 1 s long epochs starting at 100 ms before the onset of an eye-fixation. Fixation onsets are timestamps marking the beginning of fixations. These onsets were obtained by subtracting 100 ms from the moment a fixation which was identified using the method detailed above. As described above, this timestamp was then compared to EEG timestamps to locate the start of the FRP. As mentioned before, there are inaccuracies in this comparison of around four samples (or around 16 ms). These imprecisions are however inconsequential for considered analysis as highly accurate latency is not needed. The average ERP profile of these epochs reveals a prominent peak occurring around 90–100 ms after fixation onset (as seen in Figure 7A). This peak, termed P100, is present in occipital EEG channels (see Figure 7B) and commonly associated with natural fixations (Herrmann et al., 2005). According to Luck (2014), the amplitude of this early potential provides an indicator of the subject's current attentional effort: a higher amplitude of this peak correlates with more effort used for the processing of visual information. So far, these properties have been investigated in very controlled studies and rarely in closer to everyday-life visual search tasks or BCI settings.

To investigate the utility of this peak's amplitude for BCI, we investigated its correlation with changes in the presented stimuli needs to be verified. This early peak was therefore identified and located in each constructed epoch and its amplitude extracted accordingly. This was done by filtering the epochs (using 1–10 Hz bandpass filter), keeping only prominent oscillations in the signal segment intact, and employing a Multiple Linear Regression with dispersion term (MLRd) (Hu et al., 2011; Wobrock et al., 2016) on the first 200 ms of each epoch. Single epochs usually contain certain amounts of noise which make the location of specific potential peaks difficult. The MLRd algorithm consists of creating regressors for a peak, present within a reference signal segment, as to locate this same potential within similar signals. These regressors consist of the principal components of the concerned reference potential and are constructed in such a way to account for possible shifts in peak amplitude, latency (i.e., temporal location in the epoch) or morphology (i.e., shape of the peak) in an epoch. Fitting the regressors onto the signal then allows to locate the concerned peak reliably within every epoch. This includes epochs in which peaks are occluded or difficult to differentiate from other ones. Furthermore, the MLRd also allows to locate peaks accurately when slightly inaccurate fixation onsets are presents. However, in such cases, more indepth analysis of peak latency should be avoided. This method is explained in greater detailed in Wobrock et al. (2016) as well as in the Supplementary Material. Here, only the peak at 90–100 ms contained within the Oz Channel recording was located and is further investigated in the following.

**Figure 8:**
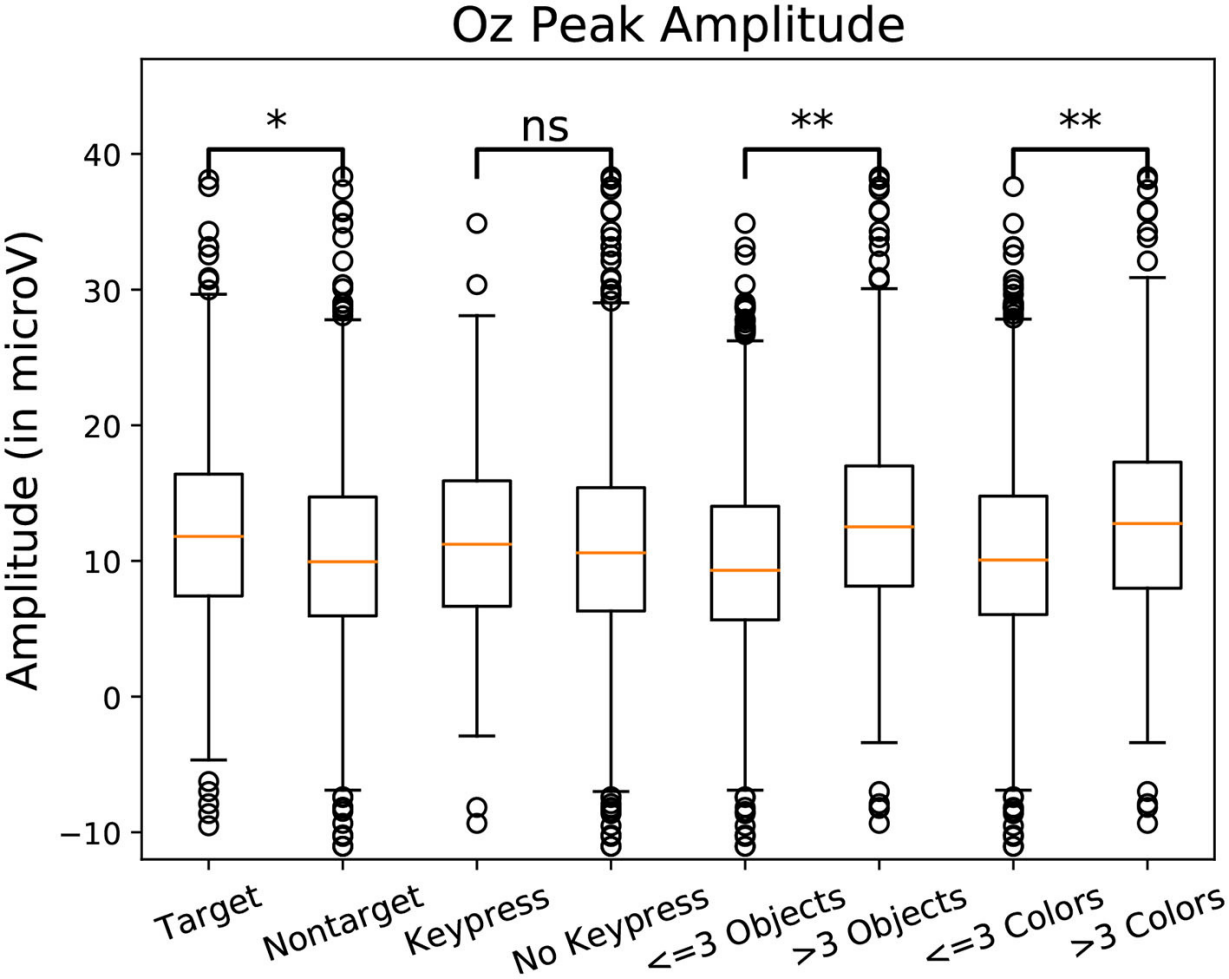
Amplitude of the P100 component peak measured at the Oz electrode measure for one subject. Amplitudes were grouped by class. Eight categories were compared in pairs. Target/non-target define if a target object was fixated. Keypress/No Keypress is determined based on the participant performing a keyboard press (the space key) during fixation. The categories labeled Objects are defined depending on the amount of shapes in the foveal region. The categories labeled “Colors” are defined depending on the amount of different colors in the foveal region. Significant differences are present between 3 pairs of categories. Using Student *t*-test: “ns” means *p*-value > 0.05, “*” means *p*-value < 0.05, “**” means *p*-value < 0.01.

To observe the variations in peak amplitude across attentional demand, epochs and the MRLd-extracted amplitude of their early peak were grouped according to the visual content of their corresponding fixation region. For each fixation, the contents of the corresponding visual region was examined and categorized. This was done depending on the number of objects, the amount of different colors, the presence of target objects and the presence of keyboard presses during the fixation. It is to note that two objects were considered different in color when the value of Equation (1) was above 200. The amplitudes were then compared between categories (as seen in Figure 8). This was shown in Figure 10 where each significance comparison bracket indicates a pair of complementary amplitude distributions.

**Figure 9:**
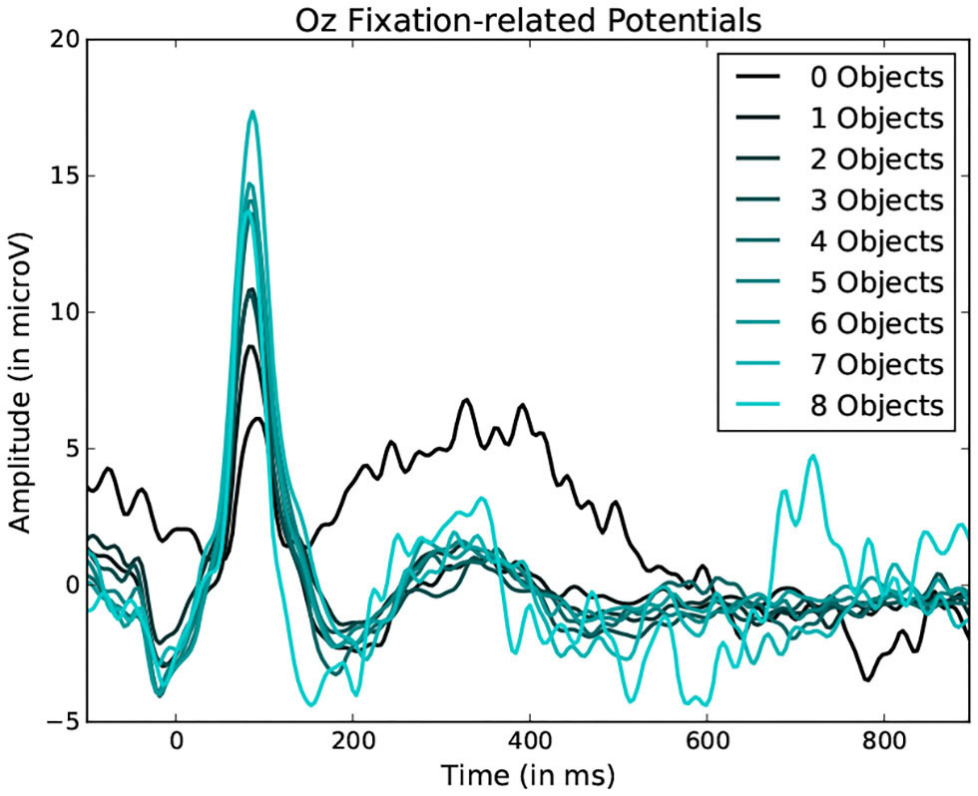
Average fixation related potential epoch profiles recorded on the Oz electrode. The lighter the color, the more objects were present within the fixated region. Data from one participant was used in this figure.

**Figure 10:**
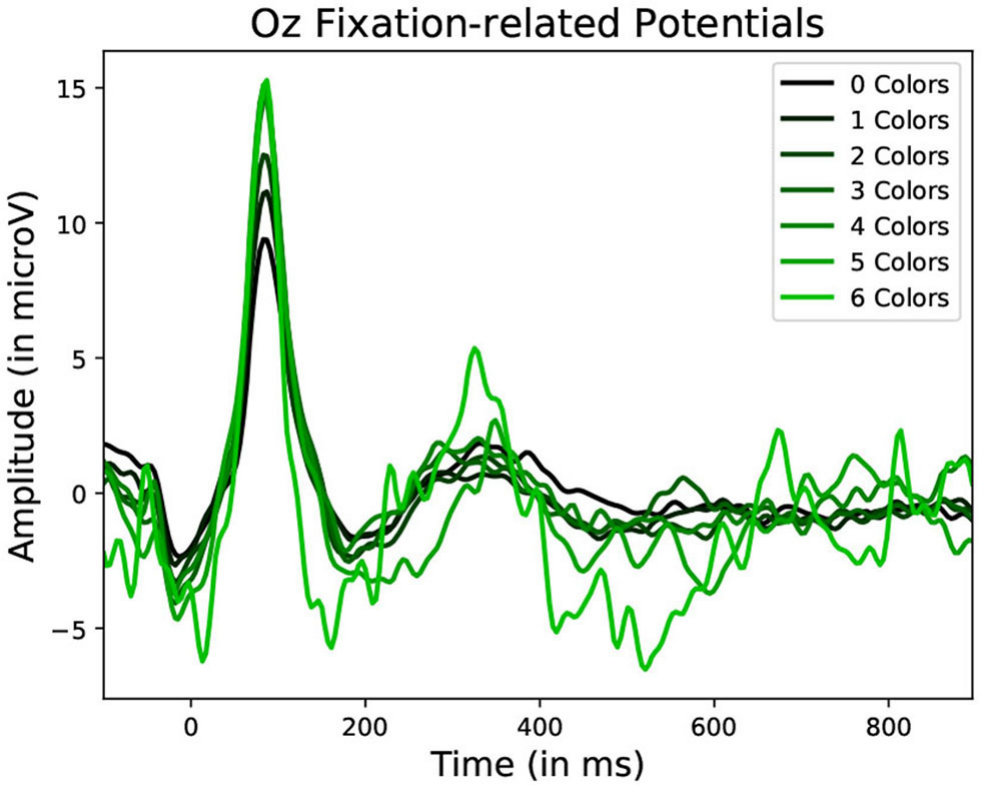
Average Fixation related potential epoch profiles recorded on the Oz electrode. The lighter the color, the more different colors were present within the fixated region. Data from one participant was used in this figure.

Statistically significant differences in P100 peak amplitude are present between conditions of different number of objects and colors within the foveal area of the fixation (as per two tailed *t*-test, *p*-value < 0.01 in both cases). These differences are present in all subjects. Additionally, a higher number of objects or colors in the fixated region correlates with in higher peak amplitudes being measured (as qualitatively observed in Figures 9, 10). This indicates that the P100 amplitude can be interpreted as correlated to the amount of visual information to process. Interestingly, a statistically significant difference (*p*-value < 0.05) in peak amplitude is also present between a fixation on target and non-target object (seen in first pair of boxplots in Figure 8). This suggests that while peak difference mostly relate to the quantity of information, the relevance of this information in the subject's current task also has an influence on peak amplitude. Variation between subjects regarding the significance of this property is, however, large. Indeed, certain subjects did not present significant difference which may reflect their of a individual strategy for visual exploration (Nikolaev et al., 2016).

**Figure 11:**
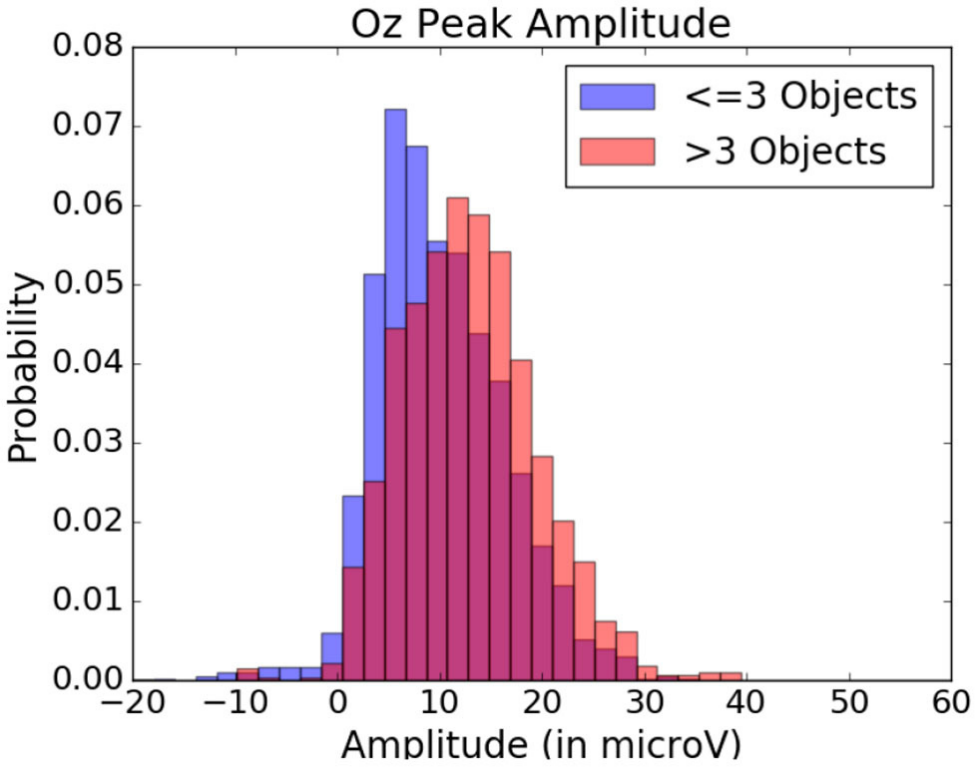
Peak amplitude variation depending on the quantity of objects within the visual fixation region. Data from one participant was used in this figure.

These amplitude differences can, however, only observed reliably over a larger set of epochs: as peak amplitude presents strong variation even between fixations on the same visual regions, the epochs from multiple visits have to be averaged for this property to emerge clearly. This is especially necessary as amplitude distributions of each of the considered categories strongly overlap (see the standard deviations of Figure 8 shown more prominently in Figure 11). As such, the attentional cost of processing a visual region is only attainable when multiple visits of a same location are performed. In their current form, these single trial FRP evaluations may still allow to investigate if certain regions of the interface require more attentions from the participant. As such, the method allows to gain general hints of the visual difficulty a participant is experiencing. These hints can then be used by designers to improve the concerned system and its interface.

The information obtained through this FRP method is similar to the one gained through gaze pattern analysis of eyetracking but can provide information faster. So far, these insights provided by the proposed methods mainly concern passive interaction (i.e., exploration) of the interface. To inspect the value of this bi-modal setup further, properties relating to the active manipulation of the interface must be looked at in greater detail. Looking at fixations still remains useful as they represented concrete steps of communication with the interface which can allow for deeper understanding of interaction than just time-locked FRPs. As, rather than being restricted to 1 s of signal, fixations can be inspected over their full duration.

### 4.3. Analyzing Entire Fixations

Fixation-related potentials can be observed during any manipulation where eye-movements occur. Commonly, in many interaction contexts, the recorded EEG signal will also contain other interaction-relevant components unrelated to FRPs. These ERP components usually only manifest themselves when subjects encounter specific tasks or stimuli (Teplan et al., 2002), making it difficult to utilize these components consistently in a broad variety of situations. Similarly, ERP are difficult to utilize when they can only be labeled with vague user-side information (e.g., fixation duration). Nonetheless, depending on the studied interaction, being able to still access these potentials increases the likelihood of gaining insights into underlying nature of the interaction. A notable example of this are Error-related Potentials, which occur when a subject makes a manipulation error or obtains an unexpected outcome (Nieuwenhuis et al., 2001).

**Figure 12:**
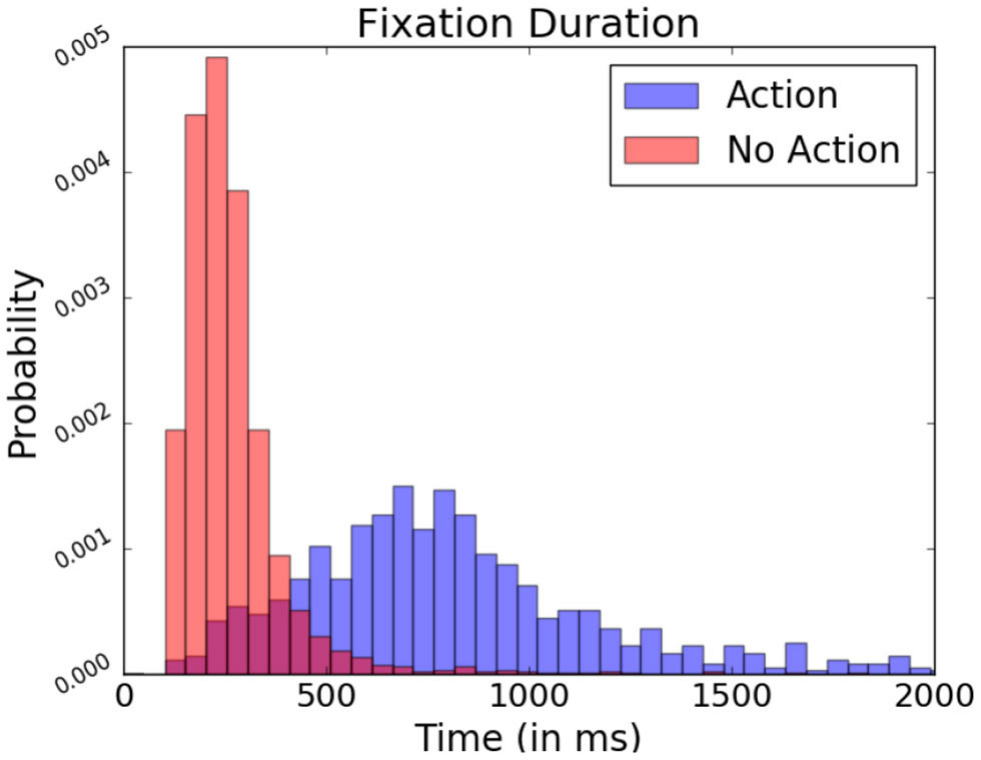
Relative fixation duration depending on the present or absence of keyboard presses, expressed in probability. Data from one participant was used in this figure.

As mentioned previously, in FRP studies, fixation onsets serve as contextual reference points when analyzing the EEG signal. Non-fixation-related events are not time-locked within fixations, making their observation through these reference points more difficult. However, events which provoke these potentials often require specific focus from the part of the subject (Hayhoe and Ballard, 2005). In the present study, subjects performed keyboard inputs to select target objects. When locating these actions in relation to the subject's fixations, fixations containing a keyboard press presented a generally longer duration occurred than those where it did not contain presses (as seen Figure 12). This difference in fixation length serves as a means to identify moments in which more complex interaction may have happened.

However, there is a considerable overlap in fixation duration between “exploratory” non-action fixations and “input” action fixations during which the participant actively manipulated the system, rendering it difficult to spot other event-related potentials difficult through this fixation duration measure alone. To improve their detection, data from EEG recordings ranging over the entire duration of a given fixation is probed rather than fixed 1 s segments. Ensuring the varying fixation duration does not bias signal analysis, the relative bandpower of these EEG segments is examined. Relative frequency band power has been traditionally used to assess the wakefulness or different states of arousal of the observed subject (Benca et al., 1999). Here, however, it allows identification fixation which contains potentials as characterized by prominent low frequency waves in the EEG signal. The studied bands are the delta (0.5–4 Hz), theta (4–8 Hz), alpha1 (8–10 Hz), alpha2 (10–13 Hz), and beta (13–30 Hz) bands. They are calculated via Fourier Transformation and are expressed proportionally to each other.

**Figure 13:**
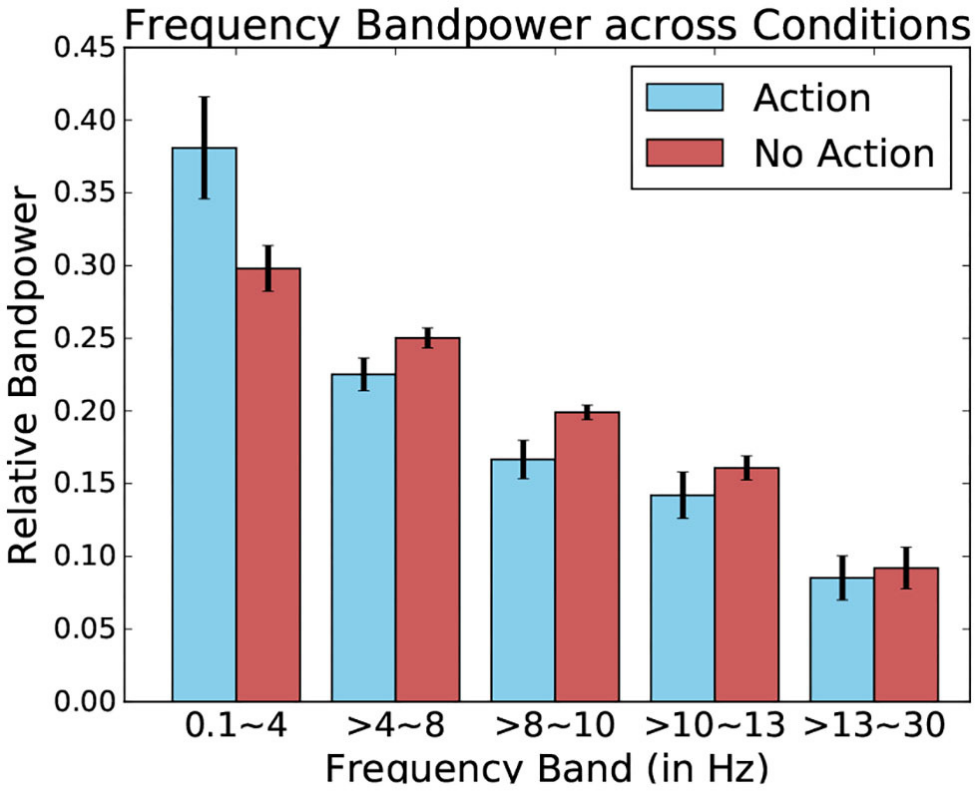
Relative frequency bandpower depending on the presence or absence of keyboard presses. Data from one participant was used for this Figure.

When comparing these relative bandpowers across conditions, major differences present themselves between the fixation-bound EEG segments which contained keyboard pressed and those that contained no such events (this can be seen in Figure 13). Indeed, this difference is highest when comparing low-frequency delta bands. This band contains a much higher percentage of the total power of the signal when observing fixation segments containing keyboard presses, suggesting the presence of low frequency potentials.

**Figure 14:**
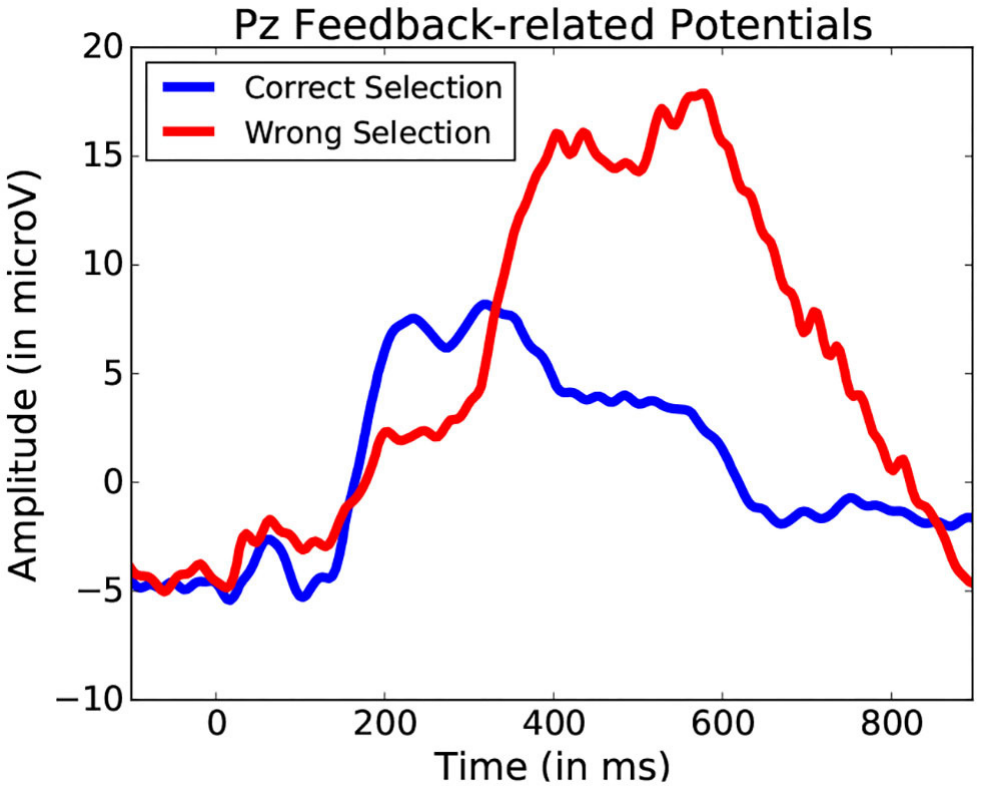
Average potentials occurring at the onset of a key press performed to validate shape selection. Data from one participant was used for this Figure.

Using these five bands as features for each fixation-bound EEG segment (i.e., fixations), the single-trial discrimination between both conditions (i.e., action and non-action) is tested. This is done using FDA as a classification algorithm. A five-fold training of this method resulted in an average accuracy of 83.11% (±4.71) across all subjects between action and no-action segments. This confirms that the actions performed by the user produce different activity which is allows to quite accurately discriminate between passive and active interaction. While the keyboard inputs imply the presence of the Event-related desynchronization (Pfurtscheller and Da Silva, 1999), which could serve as a main discriminative feature for this classification, other event related potentials could also factor into this differentiation. One example of such potentials could be Error-related Potentials (as the averaged ERPs recorded after keyboard input represented in Figure 14 suggest). In either case, these frequency bands values allow to identify the presence of moments of more active interaction.

This method offers greater clarity than the simple fixation duration comparison. Yet, it still does not allow for an exact event-related potential location, and thus the exact location of the start of the event remains unknown without using system-side information. A still finer analysis of fixation segments should be considered in the future to improve localization. While it may improve detection, such an approach would still not always identify the actual event onset required for proper ERP analysis. Figure 14 illustrates a situation where target and non-target shape selection results in similar yet slightly shifted signal profiles. The FDA classification method permits to narrow down and interpret other events that occur during manipulation. This allows for a general detection of a large variety of events without adapting user or system side components, providing better understanding of the importance of certain specific moments during interaction. Further analysis specific form the explored interface can be considered from there on.

## 5. Discussion

The goal of this work was to offer a way to explore how a bi-modal BCI, combining EEG and Eyetracking recordings could be used to give access to valuable information about an interaction without relying on system-side information. Three methods were developed in this work revealing insights about general task difficulty, localized increases in attentional effort and identification of different moments of interaction. Our three approaches build almost exclusively on information from user-side components, relying little on system-side information thus decreasing necessary BCI system adaptation requirements and increasing its portability to new scenarios. The proposed methods were tested in a sedentary setting but could technically be extended to a mobile scenario. This may, however, require a finer tuning of modalities to account for new artifacts and tackling of technical challenges induced by user motion.

A major question that remains is if the information provided by these three perspectives are both sufficient and stable enough to propose an interesting use of Brain-Machine interfaces to assist in the inspected human-machine interaction. Within the conducted study, the first results of these methods are promising and novel when compared to classical approaches.

With the first method, a more general investigation of the interaction was proposed by utilizing the bi-modal FRP setup. The two core recording modalities were used separately accessing their respective informative value about the interface. Namely, this includes the analysis of gaze patterns and potentially the observation of relative frequency band analysis over the entire interaction. Here, the inspection of gaze pattern, especially when considering relative fixation duration, region revisits and saccade length, permits an initial estimation of the difficulty a participant encounters during a given task. More specifically, gaze patterns help to delineate between features of the scene exploration which can be used to infer the origins of difficulty. When considering interface analysis, such insights allow to understand which characteristic of the presented scene introduces difficulty. However, in this particular case, these differences, in particular relating to saccade distance could be related to the spatial organization of the stimulus scene.

The second method focused on identifying attentional cost in FRPs. This was done by observing early P100 peak in the FRP epochs. Significant increases in P100 peak amplitude were present when regions presenting higher quantity and relevance of information were observed. Observing the properties of individual potentials opens a way for a more in-depth analysis on the importance of specific fixations during exploration and the visual information they considered. This approach could provide information for identifying which locations are most relevant for the subject, as well as which moments required more attention. The method, however, also revealed a strong variability of early potentials which make their properties only reliably accessible using a larger set of epochs. To expand the use of this property to function with a smaller number of fixations, an algorithm using finer heuristics or additional reference points than solely the P100 amplitude can be considered. Furthermore, the correlation of amplitude with attentional cost could, for example, enhances gaze pattern analysis, allowing for the construction of a more detailed visual heatmap closer to the subject's implicit perception of the scene. While far from perfectly accurate, this may allow to identify more locally when the user is having difficulties.

The last method was used to identify more active moments of interaction. Fixation-bound epochs were be used as an alternative way to segment the EEG signal. The resulting segments follow the individual fixations the participant performed, which add flexibility to the analysis of the EEG signal when compared to the stricter 1 s FRP epochs. As fixations vary in length, these segments cannot be traditionally used for classification, like FRP epochs, but can serve as orientation points to access other events that also occurred during interaction. Relative frequency bandpower analysis was used to differentiate quite accurately between fixations. Practically, this would augment analysis of interfaces further, giving information on when events occurred and what region was fixated at these moments of interaction. One could consider using this method for an even more precise analysis on the obtained frequencies. Small variation in these relative event-related frequencies could, for example, be utilized to identify and differentiate between different types of potentials. This could notably be employed to identify error-related potentials (Spüler and Niethammer, 2015), thus giving a deeper understanding on how the subject handles the interface.

All of the three presented methods rely exclusively on data gathered from EEG and Eyetracking. Furthermore, as all underlying paradigms occur naturally during regular interaction, this approach to interface evaluation is flexible and can be adopted by a wide range of visual interfaces. The obtained results show that proposed methods, while not offering perfect precision, still provide meaningful information about peaks on a single trial level. These methods thus provide new information on the user's state without requiring additional adaptations on the BCIs system-side or imposing new tasks on the subject. Additionally, these methods allow to gain a holistic view of the interaction by obtaining general and momentary information about the interaction. Notably, by concentrating on momentary FRPs, a much more targeted analysis of the interaction becomes possible, and by focusing on EEG and Eyetracking alone, general difficulties are identified. This characterizes our multimodal approach as being more resource efficient than classical BCI approaches, which traditionally rely on a rather narrow analysis of specific and properly labeled events.

## 6. Conclusion

In conclusion, the three presented methods offer a novel and useful way to analyze interfaces. They integrate themselves easily into BCI and are portable between scenarios. Our approach permits to obtain more information from the user side for analysis, minimizing the necessity of system-side adaptations and the workload they require. The methods themselves offer new windows into aspects of interaction allowing analysis on new and different levels. This ranges from a general overview of the task, to identifying very specific moments of visual attention or active interaction. However, all these described approaches, while usable, require testing as well as further improvement to be effectively used and efficiently implemented to reveal their full potential when applied in a live BCI setting. Further improvements could also be obtained by focusing on information contained within the, so far mostly explored, short fixations. The average fixation lasts 250 ms and over 70% of fixations last under 300 ms (Hooge et al., 1998), which remains true in the presented study (see Figure 12). Looking at other paradigms, interaction relevant properties are known to occur later in the epoch (e.g., P300, N400), signifying that important information is potentially present but overlapped by following fixation causing artifacts (Woldorff, 1993). Correcting this overlap may allow an even further expansion of this system. Further research is necessary to explore all mentioned possibilities.

## Data Availability Statement

The datasets presented in this study can be found in online repositories. The names of the repository/repositories and accession number(s) can be found below: Name: Wobrock D. Dataset Exploring Fixation-related Potentials in Naturalistic Search Tasks. Bielefeld University; 2020. https://pub.uni-bielefeld.de/record/2943918.

## Ethics Statement

The experimental procedure and written consent form for this study were approved by the ethics committee at Bielefeld University. The patients/participants provided their written informed consent to participate in this study.

## Author Contributions

DW and AF designed the study and analyzed the data. DW collected the data. HR and TS supervised the project. All authors wrote and revised the article.

## Conflict of Interest

The authors declare that the research was conducted in the absence of any commercial or financial relationships that could be construed as a potential conflict of interest.
