# Supplementary material for: Using Fixation-Related Potentials for Inspecting Natural Interactions
Source: Front Hum Neurosci. 2020 Nov 5;14:579505. doi: 10.3389/fnhum.2020.579505 (PMC7674802; doi:10.3389/fnhum.2020.579505)
Supplement: Supplementary file 1 [file Data_Sheet_1.PDF]

Appendix
to
Using Fixation-Related Potentials
for Inspecting Natural Interactions

Multiple Linear Regression with dispersion term (MLRd) algorithm and ERP property extraction

The Multiple Linear Regression with dispersion term allows for the location of potentials observed in EEG Grand Averages within individual single trial epochs.

MLRd algorithm (see below for graphical representation):

Consider a set of single trial epochs, where each epoch has a length of N samples.

1. The grand averages of each recorded EEG channel are created by averaging all epochs together. (A to B in the graphical representation)
2. The grand average of a chosen channel is segmented according to the inflection points of the signal, so that each segment contains a peak. (B)
3. The grand average segments containing peaks of interest are marked. Here we consider M, the number of marked segments. (B)
4. The individual epochs used to create this grand average are randomly assigned to groups of 20 epochs which are averaged together. (A to C)
5. These random averages are segmented using the same segmentation as the one determined by the grand average. (C to D)
6. For each segment that was previously marked, the corresponding random average segments are extracted, so that the values of the averaged epoch outside of the segment are set to 0. (D to E)
7. The gated average epochs of one segment are given to Principal Component Analysis (PCA) as features. It results in obtaining the first 3 principal components of a length of N sampled per marked segment. (E to F)
8. The obtained components of each marked segment appended together, resulting in a matrix R of size (3M x N). (F to G)
9. A linear regression is performed on each single trial epoch, using the matrix R as regressors. (H)
10. The fitted regressors are separated according to the peak segment they originated from and summed together (I)
11. If the expected extremum (minimum or maximum) is not present within these corresponding set of fitted regressors, the marked peak of interest is considered absent.
12. If an expected extremum is present, it‘s location is compared with the closest comparable peak in the actual epoch. (I)
13. This closest peak is then considered a single trial occurrence of the potential of interest.

Peak Property extraction :

Once a potential is located in a single epoch, three properties can be extracted : its amplitude, latency and morphology.

- Amplitude corresponds to the magnitude of the extremum of the peak.
- Latency corresponds to the number of the sample of the extremum in the signal segment.
- Morphology corresponds to the number of samples between the first signal inflection points left and right of the extremum.

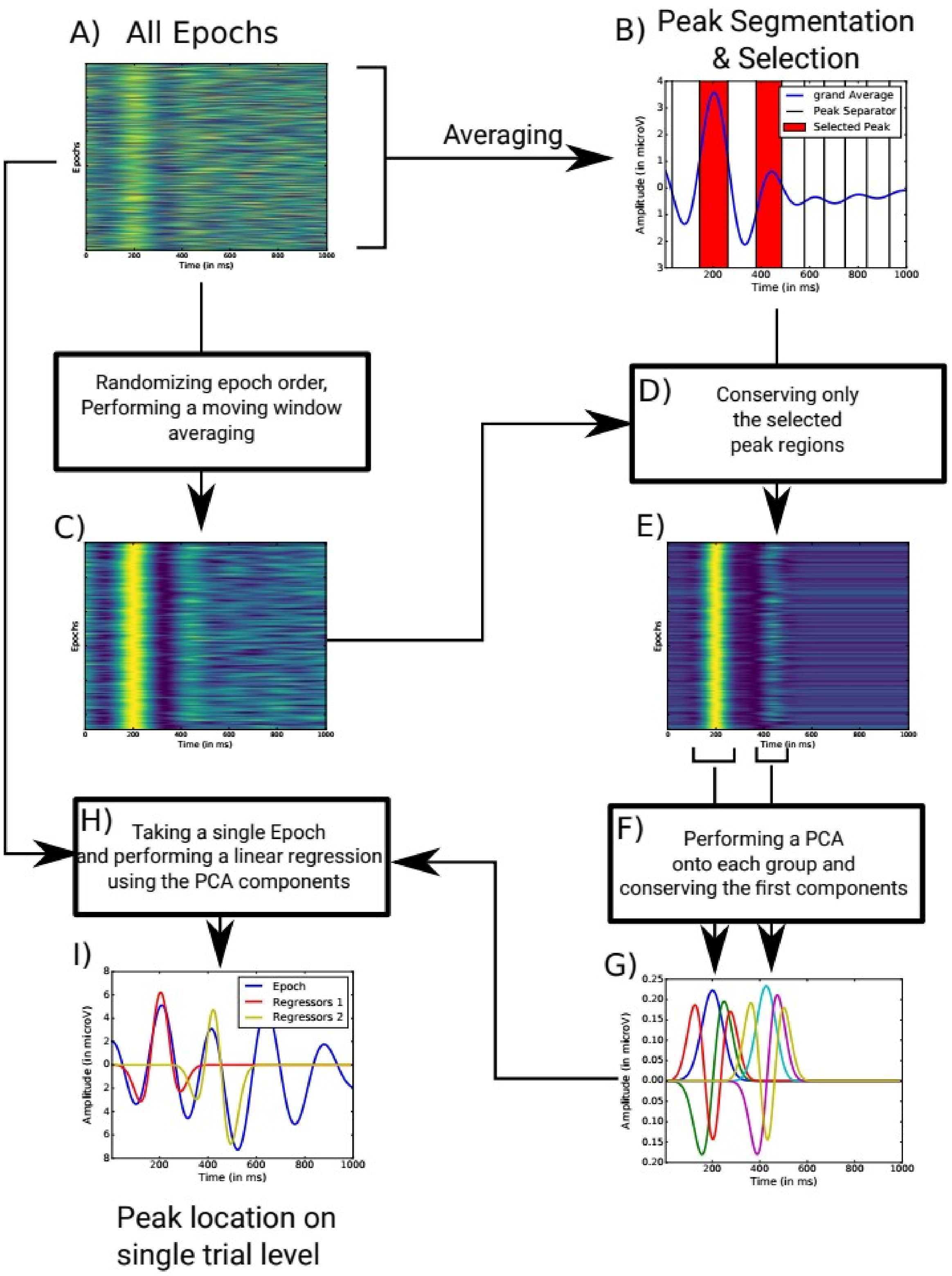

A) All Epochs
Epochs
Time (in ms)
Averaging
B) Peak Segmentation & Selection
grand Average
Peak Separator
Selected Peak
Amplitude (in microV)
Time (in ms)
Randomizing epoch order, Performing a moving window averaging
C)
D) Conserving only the selected peak regions
E)
H) Taking a single Epoch and performing a linear regression using the PCA components
F) Performing a PCA onto each group and conserving the first components
I)
Epoch
Regressors 1
Regressors 2
G)
Peak location on single trial level
